# Quantifying health utilities of young adult orthodontic patients using the time trade-off method: a cross-sectional study

**DOI:** 10.1590/2177-6709.28.2.e2321238.oar

**Published:** 2023-06-05

**Authors:** Budhaditya PAUL, Arun Srinivasa URALA, Shashidhar ACHARYA

**Affiliations:** 1 Manipal Academy of Higher Education, Manipal College of Dental Sciences, Department of Public Health Dentistry (Manipal, Karnataka, India).; 2 Manipal Academy of Higher Education, Manipal College of Dental Sciences, Department of Orthodontics and Dentofacial Orthopedics (Manipal, Karnataka, India).

**Keywords:** Health utility, Time trade-off, Malocclusion, Quality of life

## Abstract

**Objective:**

To study the feasibility of time trade-off (TTO) method in quantifying health utility ratings in different types of malocclusion.

**Material and Methods:**

In this cross-sectional study, 70 orthodontic patients aged 18 years or above, reporting for treatment/consultation, were included and interviewed. Malocclusion-related health utilities were assessed through the TTO method, and oral health-related quality of life was measured with the help of Orthognathic Quality of Life Questionnaire (OQLQ). Angle’s classification of malocclusion was recorded. Bivariate analyses and multivariate Poisson’s regression were done to find out an association between the oral health utility values, OQLQ and demographic and clinical characteristics.

**Results:**

Patients with skeletal Class III malocclusion had lower health utility values than those with Class I and Class II malocclusions (*p*=0.013). Poisson’s regression showed that Angle’s Class II division 1 (0.90, CI 0.84 to 0.97), Class III (0.68, CI 0.59 to 0.95) and Skeletal malocclusion (0.79, CI 0.71 to 0.87) and OQLQ scores (1.0, CI 1 to 1.003) were found to be significant predictors of TTO utility scores.

**Conclusions:**

TTO utilities were found to be valid and well correlated with clinical findings. Health utilities could serve as useful and reliable markers of health-related quality of life (HRQL) among individuals or communities and help cost-effective preventive or intervention programs planning.

## RESUMO

**Objetivo:**

Estudar a viabilidade do método *Time trade-off* (TTO) para quantificar escores de valoração da saúde em diferentes tipos de má oclusão.

**Material e Métodos:**

Neste estudo transversal, foram incluídos e entrevistados 70 pacientes ortodônticos com idade igual ou superior a 18 anos, que compareceram para tratamento/consulta. A valoração da saúde em relação à má oclusão foi avaliada por meio do método TTO e a qualidade de vida relacionada à saúde bucal foi medida com a ajuda do Questionário de Qualidade de Vida Ortognática (*Orthognathic Quality of Life Questionnaire*, OQLQ). A classificação da má oclusão segundo Angle foi registrada, e análises bivariadas e regressão multivariada de Poisson foram feitas para verificar qualquer associação entre os escores de valoração da saúde bucal, OQLQ e características demográficas e clínicas.

**Resultados:**

Os pacientes com má oclusão esquelética de Classe III apresentaram escores de valoração da saúde mais baixos do que aqueles com má oclusão de Classe I e Classe II (*p*=0,013). A regressão de Poisson mostrou que a Classe II de Angle divisão 1 (0,90, IC 0,84 a 0,97), Classe III (0,68, IC 0,59 a 0,95), má oclusão esquelética (0,79, IC 0,71 a 0,87) e os escores do OQLQ (1,0, IC 1 a 1,003) foram considerados preditores significativos dos escores de valoração pelo método TTO.

**Conclusões:**

Os escores do TTO foram considerados válidos e bem correlacionados com os achados clínicos, e podem servir como marcadores úteis e confiáveis da qualidade de vida relacionada à saúde (*health-related quality of life*, HRQL) entre indivíduos ou comunidades, e ajudar no planejamento de programas de prevenção ou de intervenção, com uma boa relação custo-benefício.

## INTRODUCTION

Health-Related Quality of Life (HRQL) is a multi-dimensional idea that includes domains related to physical, mental, emotional, and social functioning. It directly measures population health, life expectancy, causes of death, and focuses on the impact that health status has on quality of life. Quality of life is increasingly seen as an important factor in deciding resource allocation for health interventions.^1^ Utility instruments help in deriving a single number representing the preference to a particular health state.^2^ The utility scores provide the quality of life weights needed to calculate quality-adjusted-life-years (QALY) needed for economic evaluation of health-related interventions/ policies.

The indirect (or holistic) methods of utility measurement are the generic preference-based measures or disease-specific preference measures, like the Health Utility Index, Quality of Well Being Scale and EuroQol-5D. The direct methods used to measure utilities include the rating scale, the standard gamble and the time trade-off. The direct methods are more suited for utility assessments among patients/individuals suffering from a particular health condition, whereas indirect measures are usually administered to the general population. Indirect measures are simple, easy to administer and less time-consuming, but may not entirely capture the complex multifactorial conditions and the benefits of mitigating factors like family, friends, work and other influences that only a patient feels.^3^ Among the direct methods, time trade-off (TTO) method, introduced by Torrance et al.,^2^ remains one the most popular methods for directly eliciting preferences for health states. It is a choice-based technique of obtaining a health state utility, reflecting the length of remaining life expectancy that a person is willing to exchange in order to avoid living in a less than perfect health state. The underlying assumptions of the TTO method is that an individual with lower utility values has poorer health-related quality of life, and assigns a lower value (utility) to his current health status than someone with a higher TTO value.

Malocclusion is often described as a morphological variation in which teeth are present in a deviant position in relation to adjacent teeth and/or the opposing teeth upon closure of the jaws. In India, the prevalence of malocclusion ranges from 20% to 92%.^4^ ^,^ ^5^ Malocclusion has long-lasting ramifications on the psyche of an individual, and can have a negative effect on one’s self image, career progress, societal acceptance and quality of life. They can also be associated with impaired masticatory function and speech.^1^ ^,^ ^6^ In dentistry, specific HRQL questionnaires include the Psychosocial Impact of Dental Aesthetics Questionnaire (PIDAQ),^1^ Oral Health Impact Profile (OHIP),^7^ and the Orthognathic Quality of Life Questionnaire (OQLQ).^8^ The OQLQ is the only instrument that explores the subjective influence of orthodontic specific conditions in young adults.^9^

Most of the studies on malocclusion are focused on children/adolescents, as they are the key focus of orthodontics. In developing countries, however, orthodontics is still out of reach of vast sections of the population. Regular dental screening programs at schools or elsewhere are largely non-existent. The prohibitive cost of orthodontic treatment also plays a role in children neglecting orthodontic treatment at the adolescent stage. A combination of self-awareness, peer pressure and relative financial independence make them seek orthodontic treatment in their early adult years. It has been reported that adults are more affected by their malocclusion, with significantly higher emotional and social impacts than adolescents.^10^

A literature search revealed only one study where an attempt was made to measure utilities among orthodontic patients awaiting surgery to correct dentofacial deformities.^11^ Studies that measured utilities among patients with different types of malocclusion were not reported in the literature. Hence, the objectives of the present study were to assess the feasibility of time trade-off (TTO) method in quantifying health utility ratings in different types of malocclusion. It was hypothesized that the TTO method would be effective in quantifying health utility ratings in different types of malocclusion.

## MATERIAL AND METHODS

The STROBE guidelines were followed for describing the materials and methods. The study followed a cross-sectional design and was conducted for a duration of four months, from September to December 2017. Prospective orthodontic patients waiting for consultation were recruited and interviewed by one of the researchers (BP) at the Department of Orthodontics and Dentofacial Orthopaedics in Manipal College of Dental Sciences (Karnataka, India). The study protocol was reviewed and approved by the Ethics Committee (IEC:536/2017). Participants were included only after having given their informed written consent, and the study was performed in accordance with the Declaration of Helsinki.

Patients were deemed eligible for inclusion if they were above eighteen years of age, had all four permanent first molars intact, and could comprehend the English language without hindrance. Exclusion criteria were patients with ongoing or past history of orthodontic treatment or patients suffering from psychological illnesses.

Training and calibration of the single examiner (BP) was done before the commencement of the study, consisting of two days of theoretical and clinical training in diagnosing malocclusion. For calibration of Angle’s classification process, a series of ten casts with varying degree of malocclusion were utilized. The findings of the single examiner were compared to that of the expert (AU), and the inter-examiner agreement/reliability was assessed with Kappa statistics. A kappa value of 0.94 was achieved for Angle’s classification. Intra-examiner agreement was checked by duplicate examination of every 10^th^ patient during the survey process, revealing a Kappa statistic value of 0.96, denoting high intra-examiner reliability.

A thorough oral examination was initially performed, and the Angle’s classification of malocclusion was recorded. A standardized interview was carried out by the same researcher (BP), and included the recording of demographic and clinical data. In addition, the OQLQ was administered and finally the TTO utility valuation was recorded. A pilot study was conducted considering data from twenty patients. The TTO value and OQLQ scores were used to calculate the Pearson’s correlation coefficient (r = +0.38) of the pilot study sample, and the Fisher’s arctan transformation was derived to account for the skewness of data. Taking α = 0.05 and β = 0.80, the minimum sample size calculated was sixty-eight, with an additional 10% for non-response, giving the total sample to 75. 

## TIME TRADE-OFF UTILITIES

TTO was measured through a qualitative interview in which the patients were asked to consider a hypothetical scenario. The patients were told that a new treatment regimen existed that would permanently rectify the malocclusion (if any) they were suffering from. The treatment would always be successful; however, it would decrease their survival. The subjects were offered a Choice A, which was their full life span of 68 years, or Choice B, which had a lower life span but perfect oral health. The patients were given multiple tasks with varying number of years for trade-off, until they reached a value point where they were indifferent between the longer period of impaired oral health and the shorter period of full oral health. The bias was reduced by alternating between high number of years traded off, followed by low number of years until all the years in the spectrum were ultimately captured. TTO boards were used for this purpose. The following formula was then used to calculate the TTO utility value:

TTOutilityvalue=(numberofyearsexpectedtolive–numberofyearstrade−off)/Numberofyearsexpectedtolive

For example, if a patient expected to live another 50 years, and would be willing to trade-off 10 years for perfect oral health, then the TTO utility was calculated as (50 years - 10 years)/50 years = 0.80. The TTO utility value lies between 0 (willing to trade-off remaining life for perfect oral health) and 1.0 (not willing to trade-off any year). In other words, the more number of years a patient was ready to forego, the lower was the utility value. 

## OQLQ

To capture the multidimensional aspects of malocclusion and its impact on the patients’ well-being, the OQLQ^11^ scale was used as a self-administered questionnaire. It consisted of 22 questions pertaining to four4 dimensions of health: social aspects of dentofacial deformity, facial aesthetics, oral function, and awareness of dentofacial aesthetics. Each question had five responses from 1 to 4, where 1 denoted the particular problem had a high impact and 4 denoted minimum impact, with 2 and 3 in between 1 and 4. The fifth response was NA, or “Not Applicable”, and denoted the particular problem did not have an effect or did not apply. The summation of all the responses signified the overall impact of oral condition and health. The OQLQ has been previously used to assess quality of life among orthodontic patients in India^12^ and elsewhere.^13^

Statistical Package for the Social Sciences (SPSS) v. 22.0 software (SPSS Inc., Chicago, IL, USA) was used for data analysis. Demographic characteristics, clinical characteristics including Angle’s classification, TTO utility values and OQLQ scores were calculated and displayed. The main outcome variable of interest was the utility value derived from the preference-elicitation task (TTO). Bivariate analyses were done to find out association between the oral health utility values and the variables of interest (demographic characteristics, clinical characteristics including Angle’s classification, gradation and OQLQ score) with the help of independent sample *t-*test and ANOVA. The effect of independent variables (like age, sex) and quality of life variables (like OQLQ scores and types of malocclusion according to Angle’s classification) on utility values was evaluated using generalized linear models version of Poisson regression, to control for skewness and heteroscedasticity.

## RESULTS

Seventy-five eligible patients were invited to complete the entire questionnaire and survey. Five patients returned incomplete responses and were excluded. Demographic and clinical characteristics of the patients are shown in Table 1. The mean age of the sample was 20.9 years (SD 2.4 years) and 60% were females. Around 58% presented Angle’s Class I malocclusion, 27% presented Class II and the rest, Class III and other skeletal malocclusions. The skeletal malocclusion included six patients who had pronounced mandibular prognathism and one patient who had mandibular micrognathia. 

**Table 1:** Demographic and clinical characteristics of the sample.

Demographic characteristics	n (%)
Age	
Less than 20 years	23 (32.9)
20 to 25 years	43 (61.4)
More than 25 years	4 (5.7)
Sex	
Male	28 (40)
Female	42 (60)
Clinical characteristics	
ANGLE’S CLASSIFICATION OF MALOCCLUSION	
Simple, Class I crowding less than 5 mm	25 (35.7)
Class I with crowding >5 mm, deep bite and other minor related anomalies	16 (22.9)
Class II, division 1	14 (20.0)
Class II, division 2	5 (7.1)
Class III	3 (4.3)
* Skeletal malocclusions	7 (10.0)

* Skeletal malocclusion included six patients who had pronounced mandibular prognathism and one patient who had mandibular micrognathia.

Table 2 shows the mean TTO utilities and OQLQ scores according to demographic and clinical characteristics. The mean TTO utility was 0.82 (SD 0.17). The mean OQLQ score, out of 88, was 63.52 (SD 17.35). Males had poorer oral health-related quality of life scores of 73.75 (SD 19.25), as compared to 83.17 (SD 15.04) among females. However, there were no statistically significant differences between OQLQ scores when compared against different types of malocclusion. There was a statistically significant difference in the TTO utility values between the different type of malocclusions, with Angle’s Class III and skeletal malocclusion cases having the lowest utility ratings of 0.69 (SD 0.17) and 0.61 (SD 0.38), as compared to Class I with crowding (0.88, SD 0.13), Class I without crowding (0.87, SD 0.12), Class II division 1 (0.77, SD 0.22) and Class II division 2 (0.84, SD 0.11) malocclusions (*p*<0.05). There were no significant differences between TTO scores when compared against demographic variables like age and sex, at the 5% level. 

**Table 2:** Mean TTO and OQLQ scores according to demographic and clinical characteristics.

Clinical characteristics	n	Mean TTO (SD)	Mean OQLQ (SD)
Age			
Less than 20 years	23	0.88 (0.13)	66.02 (18.35)
20 to 25 years	43	0.79 (0.19)	62.19 (17.46)
More than 25 years	4	0.85 (0.11)	63.40 (8.66)
*p*-value		*p* = 0.070	*p* = 0.573
Sex			
Male	28	0.84 (0.17)	59.00 (19.25)
Female	42	0.81 (0.16)	66.54 (15.04)
*p*-value		*p* = 0.350	*p* = 0.025*
Angle’s classification of malocclusion			
a. Simple, Class I crowding less than 5 mm	25	0.88 (0.13)	67.23 (13.92)
b. Class I with crowding >5 mm, deep bite and other minor related anomalies	16	0.87 (0.12)	66.91 (18.02)
c. Class II div 1	14	0.77 (0.22)	60.57 (18.40)
d. Class II div 2	5	0.84 (0.11)	62.40 (9.77)
e. Class III	3	0.69 (0.17)	54.94 (29.67)
f. Skeletal deformity	7	0.61 (0.38)	52.91 (18.74)
*p*-value		*p* = 0.013*	*p* = 0.119
*post-hoc*		a,b,c,d > e,f	

* statistically significant, *p*<0.05.

Table 3 shows the relationship between individual domains of the OQLQ and the demographic and clinical characteristics of the patients. ‘Social aspects of deformity’ and ‘facial aesthetics’ showed a significant gender-wise difference, with males having greater impact on quality of life than females. When considering the types of malocclusion, the domain of “Awareness of facial deformity” showed a statistically significant difference, with patients having skeletal malocclusion reporting a greater impact. 

**Table 3:** Individual OQLQ domain mean scores according to demographic and clinical characteristics.

Clinical characteristics	Social aspects of deformity	Facial aesthetics	Oral function	Awareness of facial deformity
Age				
Less than 20 years	15.35 (5.2)	17.16 (5.8)	15.53 (6.8)	18.85 (3.8)
20 to 25 years	11.71 (4)	15.50 (4.8)	16.92 (6)	17.37 (5.3)
More than 25 years	12.14 (2.8)	17.33 (6.5)	15.84 (5.8)	17.54 (6)
*p*-value	*p* = 0.084	*p* = 0.27	*p* = 0.57	*p* = 0.074
Sex				
Male	14.58 (5.8)	15.62 (7)	13.57 (6)	16.49 (6.8)
Female	18.26 (4.3)	18.41 (4.8)	14.39 (3.8)	15.24 (5.3)
*p*-value	*p* = 0.045*	*p* = 0.049*	*p* = 0.071	*p* = 0.062
Angle’s classification of malocclusion				
Simple, Class I crowding < 5 mm	17.81 (3)	16.39 (2.5)	17.03 (4)	18.60 (3.8)
Class I with crowding > 5 mm, deep bite and other minor related anomalies	17.74 (4.5)	16.04 (3.8)	16.67 (3.7)	16.44 (3.3)
Class II, division 1	14.62 (6)	15.26 (5.3)	14.91 (7.8)	15.75 (8.5)
Class II, division 2	15.37 (4.5)	14.94 (6)	15.72 (3.8)	15.78 (5)
Class III	13.86 (5.3)	13.44 (7)	14.17 (4.5)	13.35 (4.8)
Any skeletal deformity	13.71 (4.5)	14.00 (3.8)	13.81 (5.5)	11.06 (4.3)
*p*-value	*p* = 0.74	*p* = 0.24	*p* = 0.11	*p* = 0.045*

* statistically significant, *p*<0.05.

Poisson regression analyses was performed for TTO scores as dependent variable, and is shown in Table 4. The type of malocclusion and OQLQ scores significantly predicted TTO utility scores. Among types of malocclusion, specifically Angle’s Class II division 1, Class III and Skeletal malocclusion were found to be significant predictors of TTO utility scores. 

**Table 4:** Predictors of TTO utilities and OQLQ scores, using Poisson’s regression.

Predictor variable	Unadjusted TTO PR (95% CI)	Adjusted TTO PR (95% CI)
Simple, class 1 crowding less than 5 mm	1 (Ref)	1 (Ref)
Class 1 crowding >5, with deep bite and other associated minor anomalies	1.07 (0.98 to 1.15)	0.99 (0.93 to 1.06)
Class II Div 1	0.96 (0.88 to 1.04)	0.90 (0.84 to 0.97)*
Class II Div 2	1.0 (0.89 to 1.13)	0.97 (0.87 to 1.07)
Class III	1 (0.84 to 1.09)	0.68 (0.59 to 0.95)*
Skeletal malocclusion	1.2 (1.03 to 1.1)	0.79 (0.71 to 0.87)*
OQLQ score	0.99 (0.99 to 1)	1 (1 to 1.003)*

Poisson regression was used, dependent variable was TTO, adjusted for age and sex. Types of malocclusion and OQLQ score were statistically significant at *p*<0.05. PR=Probability ratio.

## DISCUSSION

Utility value analysis is being widely employed to find not only patient preferences when it comes to treatment choices, but also to investigate cost effectiveness of various treatment modalities through cost utility analyses^14^ ^,^ ^15^ Demographic characteristics like age and gender were not associated with health utilities, which was in line with previous research.^16^ ^-^ ^22^ Although the quality of life scores as measured by OQLQ scores were lesser for Class III, and skeletal malocclusion as compared to Class I and II, the differences were not statistically significant. However, significant differences were found between types of malocclusion, where the OQLQ domain of “Awareness of Facial Deformity” had a greater impact among patients having skeletal malocclusion, which was in agreement with previous research.^23^

Health utility scores were lower for Class III and skeletal malocclusion, as compared to Class I and II. In other words, the patients suffering from Class III and skeletal malocclusion were ready to sacrifice more years of their life in exchange for a complete cure than those with Class I and II malocclusions. In a previous study among adolescents, those who had more severe grades of malocclusion as measured by the index of orthodontic treatment needs (IOTN) had lower utility values than those who had milder forms of malocclusion.^24^ In the present study, even those patients who had a very mild orthodontic problem (Class I with crowding < 5mm) had utility scores of less than 1. This could be due to the subjective nature of utility scoring, in which an individual may perceive a problem where none existed. This could be even more possible in an orthodontic scenario where age-related peer pressure and narcissistic tendencies are strong. OQLQ was not associated with the types of malocclusion, nor was it significantly correlated with TTO scores. However, it was found to be a significant predictor for TTO scores after adjusting for age and sex. This could be due to intra-group variations or suppression effects of malocclusion-related variables on other variables of interest.

The limitations of the study were that a very specific cohort of clinical patients were selected, hence the generalisability of the results to other patients or the general population should be done with caution. The sample size was calculated based on the sensitivity of the TTO method to detect differences between different types of malocclusion, and not on the prevalence of malocclusion in the local area. Any future studies on the general population would have to take this into account with larger sample size. It is also possible that patients may have gradually adapted to their health state and may lose their perspective of a perfect health state, thus lowering their expectations. The OQLQ was used instead of general health-related quality of life questionnaires in this study, as we felt that a disease-specific questionnaire would be more appropriate in a clinically specific sample of patients. Although the OQLQ has been used in previous Indian studies, it needs to be further validated among the general populations. Future studies could use the general health-related quality of life questionnaires to obtain population level utility ratings, as malocclusion-related utilities may be influenced by general health status as well.

In developing countries, resources allocated for the health sector are very low, hence accurate instruments must be devised to avoid unnecessary misspending of already scarce funds. High utility scores for malocclusion in the young adult population indicated a high value placed on oral health by the patients, irrespective of the type of malocclusion. Larger studies could generate more data that may be helpful for health-care administrators /health insurance companies in formulating policies for orthodontic care in general populations. Furthermore, health utility ratings like TTO could be used to calculate Quality-Adjusted Life Years for malocclusion-related health states. The time trade-off method may be useful to health planners to conduct cost utility analyses of orthodontic interventions among target populations.

## CONCLUSIONS

» TTO utilities were found to be valid and well-correlated with clinical findings.
» Utility values were lower among those patients with Class III and skeletal malocclusion.
» Health utilities could serve as useful and reliable markers of health-related quality of life (HRQL) among individuals, as well as communities, and help cost-effective preventive or intervention programs planning.
